# Lysosomal pH regulation in aging and neurodegenerative disease

**DOI:** 10.1002/alz70861_108384

**Published:** 2025-12-23

**Authors:** Aimee W Kao

**Affiliations:** ^1^ University of California San Francisco, San Francisco, CA USA

## Abstract

**Background:**

The lysosome is critical for maintaining normal protein homeostasis in cells and has been genetically implicated in the pathogenesis of neurodegenerative diseases including Alzheimer's Disease and frontotemporal dementia. Lysosomes are normally highly acidic organelles and acidic lysosomal pH is critical for efficient macromolecular breakdown and cellular homeostasis. Lysosomal pH becomes progressively more alkaline with age in model organisms and these changes may contribute to neurodegenerative diseases. Despite this, the genetic and molecular regulation of lysosomal pH setpoint remain incompletely understood. It is further unknown if preserving lysosomal acidity can conteract the accumulation of neurodenerative disease proteins such as tau and TDP‐43. To answer these questions, we have developed a best‐in‐class reporter for lysosomal pH known as FIRE‐pHLy (for Fluorescent Indicator Reporting on pH in the Lysosome.)

**Method:**

iPSC‐derived neurons were generated that express FIRE‐pHLy as well as CRISPRi machinery. Cells were differentiated into induced neurons and used for a whole‐genome CRISPRi screen for modifiers of lysosomal pH. In addition, C. elegans were generated that express both FIRE‐pHLy and an optogenetic acidifier of lysosomal pH, Acido‐pHLy.

**Result:**

First we demonstrate that even small changes in lysosomal pH can dramatically alter the ability of iNeuron‐derived lysosomes to degrade neurodegenerative proteins such as tau. The whole‐genome screen for modifiers of lysosomal pH identifed genes whose knock down either acidified or alkalnized lysosomal pH. Many disease genes were included in these groups. Amino acid metabolism was shown to be a key regulator of lysosomal pH. Using transgenic C. elegans, we show that neuronal lysosomal pH becomes more alkaline with age and chemogenetic manipulations that preserve lysosomal pH acidity extend lifespan and detoxify tau.

**Conclusion:**

These findings highlight lysosomal pH as a potential therapeutic target for Alzheimer's Disease and related disorders.

